# Preferential Initiation and Spread of Anoxic Depolarization in Layer 4 of Rat Barrel Cortex

**DOI:** 10.3389/fncel.2017.00390

**Published:** 2017-12-15

**Authors:** Elvira Juzekaeva, Azat Nasretdinov, Azat Gainutdinov, Mikhail Sintsov, Marat Mukhtarov, Roustem Khazipov

**Affiliations:** ^1^Laboratory of Neurobiology, Kazan Federal University, Kazan, Russia; ^2^INMED - Institut National de la Santé et de la Recherche Médicale, Aix-Marseille University, Marseille, France

**Keywords:** ischemia, anoxic depolarization, spreading cortical depression, barrel cortex, barrel, optical intrinsic signals, silicone probes, electrophysiology

## Abstract

Anoxic depolarization (AD) is a hallmark of ischemic brain damage. AD is associated with a spreading wave of neuronal depolarization and an increase in light transmittance. However, initiation and spread of AD across the layers of the somatosensory cortex, which is one of the most frequently affected brain regions in ischemic stroke, remains largely unknown. Here, we explored the initiation and propagation of AD in slices of the rat barrel cortex using extracellular local field potential (LFP) recordings and optical intrinsic signal (OIS) recordings. We found that ischemia-like conditions induced by oxygen-glucose deprivation (OGD) evoked AD, which manifested as a large negative LFP shift and an increase in light transmittance. AD typically initiated in one or more barrels and further spread across the entire slice with a preferential propagation through L4. Elevated extracellular potassium concentration accelerated the AD onset without affecting proneness of L4 to AD. In live slices, barrels were most heavily labeled by the metabolic level marker 2,3,5-triphenyltetrazolium chloride, suggesting that the highest metabolic demand is in L4 when compared to the other layers. Thus, L4 is the layer of the barrel cortex most prone to AD, which may be due to the highest metabolic demand and cell density in this layer.

## Introduction

The brain is highly metabolically active and particularly vulnerable to metabolic insults. During global or focal ischemia, the limited supply of oxygen and glucose causes a fall in ATP levels, arrest in sodium-potassium pump activity and depolarization of neurons in the metabolically deprived brain regions (Lipton, 1999; Somjen, 2001). Release of potassium and glutamate into the extracellular space accelerates depolarization of the adjacent neurons igniting an avalanche-like wave of collective Anoxic Depolarization (AD), which shares many common features with the spreading depression (SD) described by Leao (Leao, 1947; Nedergaard and Hansen, 1993; Somjen, 2001; Pietrobon and Moskowitz, 2014; Dreier and Reiffurth, 2015; Hartings et al., 2017). Near total collective neuronal depolarization during AD is associated with large DC shifts of the extracellular local field potential, and with an increase in tissue light transmittance as a result of cellular swelling (Aitken et al., 1999; Joshi and Andrew, 2001; Somjen, 2001). AD is an initiator of the ischemic damage and irreversible loss of activity in the ischemic core *in vivo* and oxygen-glucose deprivation (OGD) induced injury in the submerged brain slices *in vitro* (Rader and Lanthorn, 1989; Tanaka et al., 1997; Joshi and Andrew, 2001) (for reviews, Martin et al., 1994; Lipton, 1999; Dreier, 2011). Factors that increase metabolic activity such as increased neuronal activity or elevated temperature accelerate AD onset and ischemic neuronal death, whereas reduction in metabolic demand is neuroprotective against ischemic damage (Dzhala et al., 2000; Joshi and Andrew, 2001; Tyzio et al., 2006).

Different neuronal populations and structures display different sensitivities to ischemia that may involve different metabolism in different cell types (Kawai et al., 1992; Lipton, 1999). In the hippocampus, CA1 pyramidal cells display the highest vulnerability to ischemia, which correlates with the preferential initiation and spread of AD in the CA1 region of the hippocampus (Aitken et al., 1998; Basarsky et al., 1998). The neocortex also displays heterogenous incidence and propagation of SD and AD between different cortical regions and layers (Bogdanov et al., 2016; Kaufmann et al., 2017). Previous studies using slices of non-identified neocortical areas revealed AD and SD “tropism” for the superficial layers 2/3 (Basarsky et al., 1998; Joshi and Andrew, 2001; Kaufmann et al., 2017). The somatosensory cortex and particularly its whisker-related barrel region are highly sensitive to ischemia (Lin et al., 1990), and SD and AD preferentially arise in and propagate through the whisker barrel region of the parietal sensory cortex (Bogdanov et al., 2016; Kaufmann et al., 2017). However, the initiation and spread of AD across the layers of the somatosensory cortex remain largely unknown.

Here, we addressed initiation and propagation of AD induced by OGD to mimic ischemia-like conditions in slices of the rat barrel cortex. We found that AD was specifically initiated in L4 barrels and its initial front preferentially propagated along layer 4. Preferential initiation and spread of AD in L4 correlated with the most intense L4 staining with TTC, a histological metabolic activity marker. We propose that sensitivity to metabolic insult is non-uniform across layers of the barrel cortex: it is highest in L4 barrels, where AD is preferentially initiated, that may involve the highest metabolic demand and cell density in this layer.

## Materials and methods

### Ethical approval

All animal-use protocols followed the guidelines of the French National Institute of Health and Medical Research (INSERM, protocol N007.08.01) and the Kazan Federal University on the use of laboratory animals (ethical approval by the Institutional Animal Care and Use Committee of Kazan State Medical University N9-2013).

### Brain slice preparation

**Figure 1:**
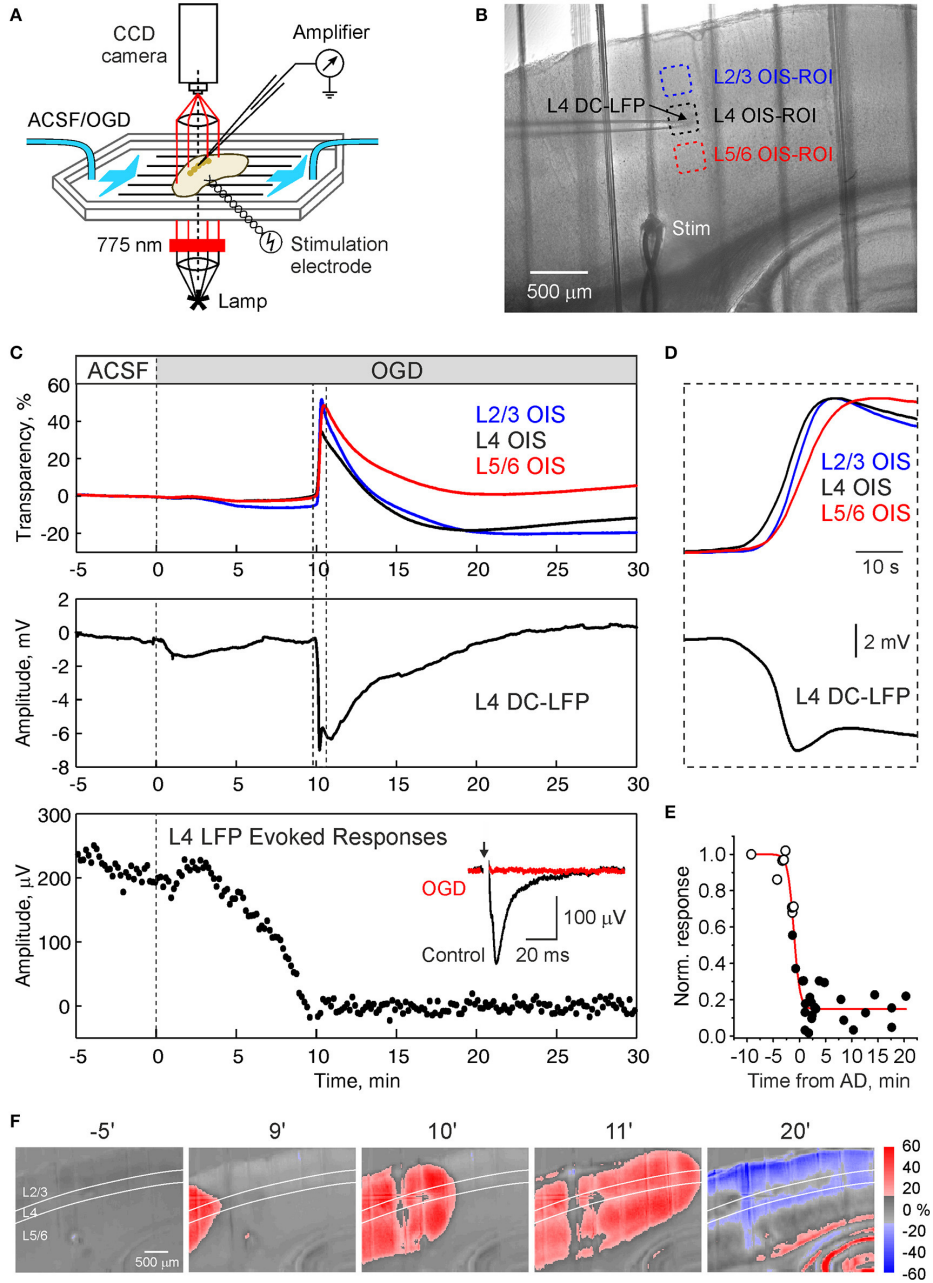
Oxygen-glucose deprivation induced anoxic depololarization in barrel cortex. **(A)** Scheme of the experimental setup. Submerged cortical slices is exposed to the ACSF in which oxygen is replaced by nitrogen and glucose is replaced by sucrose (oxygen-glucose deprivation, OGD) to mimic ischemic conditions. Local field potential (LFP) recordings and optical intrinsic signal (OIS) imaging in transmittance mode is performed to record anoxic depolarization (AD). **(B)** Microphotograph of the cortical slice. LFP is recorded from a cortical barrel, regions of interest (ROIs) in the supragranular, granular, and infragranular layers of are indicated by color boxes. **(C)** Traces of OIS from the ROIs as indicated on panel **(B)** (top), DC-LFP recordings from L4 and the amplitude of the responses in L4 evoked by stimulation of the white matter during superfusion with OGD solution. **(D)** Initial phase of AD outlined on panel **(C)** on expanded time scale. Note that onset of OIS-AD in L4 precedes the OIS in supra- and infragranular layers. Inset shows average white matter-evoked LFP response in L4 before (black) and after (red) OGD. **(E)** Dependence of the L4 evoked response recovery on the delay of reperfusion with normal ACSF from AD. Each point indicates the scalar integral of the response 15–30 min after reperfusion with oxygenated ACSF normalized to the control values. Black and open circles indicate OGD episodes with and without AD, respectively. The time values for the open circles were deduced from AD values in the experiments with repetitive OGD episodes, where each next OGD increased in duration until the AD was evoked. Red line indicates Boltzmann fit. **(F)** Snapshots of OIS at different time points after superfusion with OGD-solution. Note that the AD front preferentially propagates along L4.

Wistar rats (16–23 days old) of either sex were used. Animals were decapitated under isoflurane anesthesia (5%), the brain was rapidly removed and placed in ice-cold (2–5°C) slicing solution (modified from Dugué et al., 2005) of the following composition (in mM): K-Gluconate 140, Na-Gluconate 15, NaCl 4, EGTA 0.2, D-AP5 50 μM and HEPES 10 (pH 7.4). Four hundred μm thick thalamocortical slices were cut using a PELCO easiSlicer® vibratome (Ted Pella, Inc., Redding, CA, USA). Slices containing the barrel cortex were selected by anatomical coordinates (Khazipov et al., 2015) and the presence of barrel structures in L4 (Figure 1B). Slices were first kept in oxygenated (95% O_2_-5% CO_2_) artificial cerebrospinal fluid (ACSF) of the following composition (in mM): NaCl 126, KCl 3.5, CaCl_2_ 2, MgCl_2_ 1.3, NaHCO_3_ 25, NaH_2_PO_4_ 1.2 and glucose 11 (pH 7.4) for 30 min at 32°C and then at room temperature (20–22°C) for at least 1 h before use. For recordings, slices were placed into a submerged chamber and superfused with oxygenated ACSF at 30–32°C at a flow rate of 10 ml/min. Oxygen/glucose deprivation (OGD) was induced by superfusion with ACSF in which N_2_ replaced O_2_ and sucrose replaced glucose at equimolar concentration.

### Electrophysiological recordings

Extracellular recordings of the local field potentials (LFP) were performed in the barrel cortex using single or 16-site electrodes. Single site glass pipette electrodes were pulled from borosilicate glass capillaries (BF150-86-10, Sutter Instrument, Novato, CA, USA) and had resistances of 2–3 MΩ when filled with ACSF. Electrodes were connected via chlorided silver wire to the headstage of a MultiClamp700B patch-clamp amplifier (Axon Instruments, Union City, CA, USA). Recordings were performed in voltage-clamp mode and currents were inverted and voltage calibrated using 5 mV steps. 16-channel recordings were performed using Menendez-de La Prida style 16 shank silicone probes with a separation distance of 100 μm between electrodes (NeuroNexus, Ann Arbor, MI, USA). The signals from extracellular recordings using silicone probes were amplified and filtered (1,000×; 0–9 kHz) using a Digital Lynx SX amplifier (Neuralynx, Inc., Bozeman, MT, USA), digitized at 32 kHz and saved on a PC for *post-hoc* analysis. Stimulating bipolar electrodes were placed in the white matter or L6 above the recorded cortical column. Voltage pulses (10–50 V, 50 μs duration, 0.1 Hz) were applied to evoke LFP responses of 100–300 μV in L4.

### Optical intrinsic signal recordings

Optical intrinsic signal (OIS) recordings were performed using slice transillumination as described in Aitken et al. (1999). The slice was illuminated by a halogen lamp with a 775 nm bandpass filter and visualized using a BX51WI upright microscope equipped with a 4×/0.10 Plan N objective (Olympus, Tokyo, Japan). Images were acquired using a QIClick-R-F-M-12 CCD camera (QImaging, Surrey, BC, Canada) usually at 174 × 130 pixel resolution and 5 frames/s acquisition rate. In some experiments a higher resolution of 348 × 260 or 696 × 520 was used.

### TTC-staining

Brain slices were stained with 1% TTC (2,3,5-triphenyltetrazolium chloride) in phosphate-buffered solution (PBS) for 2–3 min at 38°C. Then slices were rinsed in PBS (for 1 min, 3 times). Microphotographs of TTC-stained slices were obtained using a SZX16 wide zoom stereo microscope equipped with a SDF PLAPO 1 × PF objective and SZX2-ILLT LED transmitted light illumination base (Olympus, Tokyo, Japan). Images were acquired at 0.7× -1× magnification using a XC50 CCD camera (Olympus, Tokyo, Japan) at 2,576 × 1,932 pixel resolution.

### Data analysis

Data were analyzed using custom-written procedures in Matlab (MathWorks, Inc., Natick, MA, USA). OIS was calculated using the first-frame subtraction approach: OIS(t) = (I(t) – I_0_)/I_0_, where I(t) – pixel intensity at the moment t, I_0_ – time-averaged pixel intensity in the preconditioned baseline period (100 s). Resulting frames were filtered with a 10 × 10 median filter. Regions of interest (ROI) were selected as square areas near recording sites. OIS traces were calculated as the average OIS signal within selected ROIs.

LFP signals were downsampled to 1 kHz. Continuous running line fit was removed using local linear regression in 300 s windows with a 10 s overlap [*locdetrend* function from the Chronux toolbox (http://chronux.org/)]. Amplitude of the LFP evoked response was calculated as a negative peak value of the LFP in the 100 ms after the stimulus relative to baseline level (average of the LFP in the 10 ms before the stimulus).

Data were smoothed by the 1,000-point moving average filter and the first derivatives were calculated. Local negative peak time of the first LFP derivative was calculated within the 20 s window preceding the negative AD peak. The value of the LFP at this time was taken as 100% and the previous time corresponding to 30% considered as AD onset. Velocity of vertical AD propagation was calculated from onset values as a distance between neighboring recording sites (100 μm) divided by AD onset delays between corresponding channels. The baseline level was calculated for each recording site as the mean value of the LFP in the −20 to −10 s time window preceding AD onset. AD amplitude was calculated as the maximal negative LFP peak from the baseline. Data from different slices were aligned by L4 and average amplitude and onset depth profiles were calculated. Depth profiles of amplitude were smoothed by the 2-point moving average filter. OIS onsets and amplitudes were calculated in same manner.

Microphotographs of TTC-stained slices were analyzed as follows. Pixel intensities were calculated along the barrel cortex and along the perpendicular direction intersecting the barrel and averaged in a 100 μm wide bar. Intensity was converted to a percentage (intensity value of each pixel divided by maximal intensity). Staining efficiency (opacity) was calculated as the value inverse to the calculated intensity.

### Statistical analysis

Statistical analysis was based on the nonparametric Wilcoxon (paired samples) or Mann-Whitney (independent samples) signed rank sum test with the significance level set at *p* < 0.05. Results are given as means ± SEM.

## Results

### Electrophysiological and optical intrinsic signals during anoxic depolarization

In the present study, we explored spatial-temporal dynamics of the OGD-induced AD in slices of the barrel cortex using extracellular recordings of LFP, and OIS recordings (Figures 1A,B). AD was initiated within 6–13 min (9.5 ± 0.5 min; *n* = 17 slices from 8 rats) and manifested as a sharp increase of light transparency attaining 29.2 ± 3.0% dI/I and negative LFP shift of 8.9 ± 0.6 mV in L4 (*n* = 17; Figure 1C). LFP signals were typically biphasic with an initial sharp negative transient followed by a secondary negative wave. The increase in OIS during AD started in L4 and further spread to L2/3 and L5/6 (Figure 1D). The responses evoked in L4 by stimulation of the white matter or L6, progressively decreased during OGD and were completely and irreversibly abolished during and after AD (Figure 1C), while the evoked responses could recover following shorter OGD episodes without AD (Figure 1E) that is in keeping with the results of previous studies (Rader and Lanthorn, 1989; Tanaka et al., 1997; Joshi and Andrew, 2001). In the experiment illustrated in Figure 1, OIS recordings revealed that AD unilaterally propagated through the slice with the leading front in L4 and delayed fronts in the supra- and infragranular layers (Figure 1F). The increase in light transmittance was followed by a decrease in light transmittance probably reflecting cellular swelling followed by dendritic beading (Aitken et al., 1999; Joshi and Andrew, 2001; Somjen, 2001). After AD in the barrel cortex, AD was also observed in the hippocampus and striatum after a several minute delay (Figure 1F).

### Vertical AD propagation in a cortical barrel column

**Figure 2:**
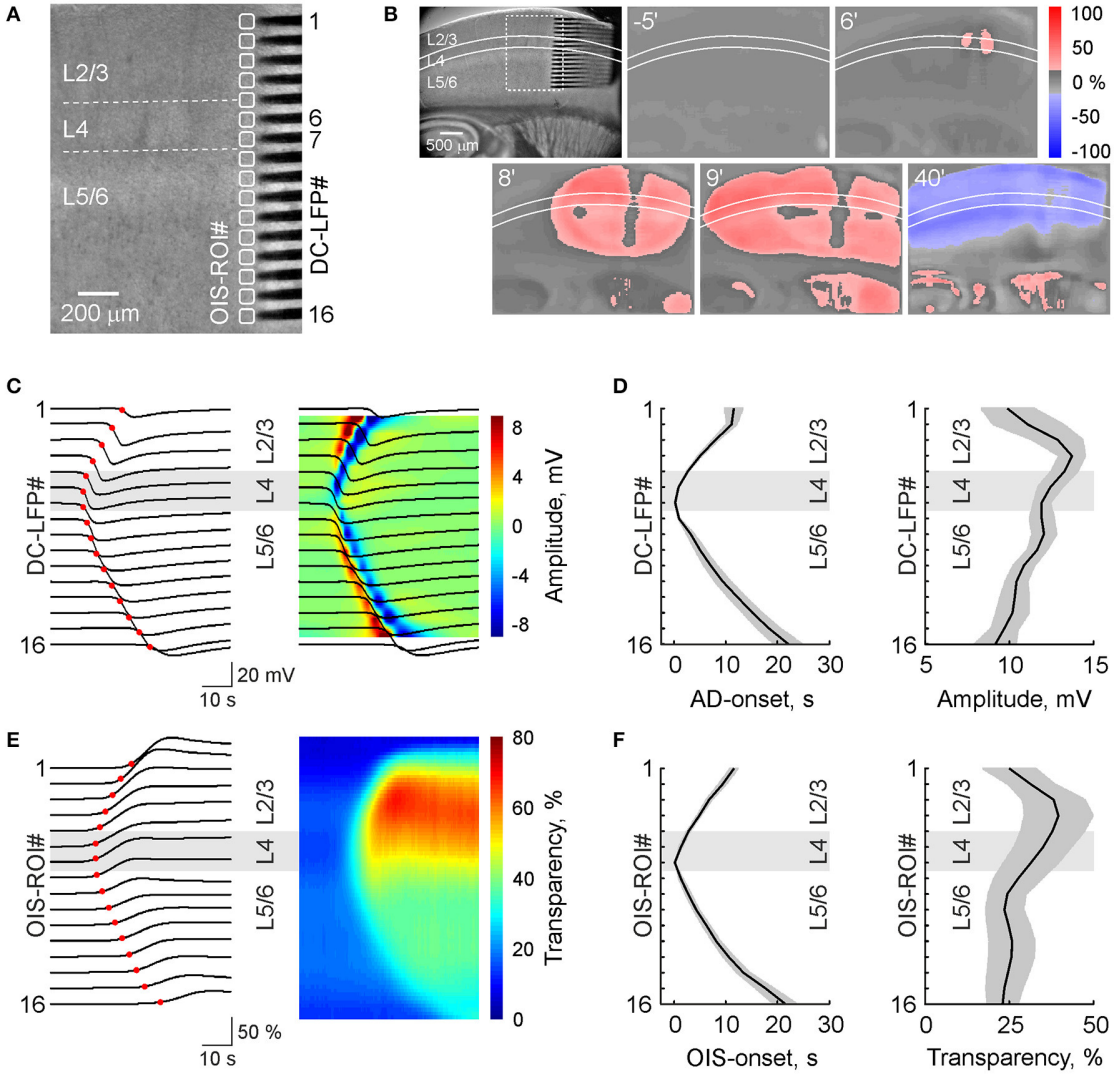
Spread of anoxic depolarization within a cortical barrel column. **(A)** IR-DIC microphotograph of a slice of the barrel cortex with a 16-shank silicone probe (100 μm electrode separation distance) vertically placed to record DC-coupled LFP from all layers of a cortical barrel column. Corresponding OIS-ROIs are outlined by white boxes. **(B)** Microphotograph of the brain slice shown on panel **(A)** on a reduced scale (left) and snapshots of OIS at different time points after OGD induction. **(C)** Corresponding DC-LFP traces (black) during AD propagation at different depths of the cortical column with the AD onsets marked at each channel with a red circle (left) and the same DC-LFP traces overlaid on the color-coded current-source density map (right). **(D)** Group data on OGD-induced AD onsets and AD amplitudes as a function of cortical depth (mean ± *SE, n* = 7). **(E)** Concomitant OIS recordings of AD (left) and color-coded transparency map calculated from OIS traces (right) in the ROIs indicated on panel **(A)**. Red circles indicate the OIS-AD onset. **(F)** Group data on the onsets and amplitudes of OIS associated with OGD-induced AD as a function of cortical depth (mean ± *SE, n* = 7). Note that AD first occurs in L4 and further spreads to L2/3 and L5/6 and that AD amplitude is maximal in L2/3.

We also performed simultaneous OIS and multisite LFP recordings from a cortical barrel column using 16-shank silicone probes (Figures 2A,B). In keeping with the results described above, AD was initiated in L4 and spread to L2/3 and L5/6 with a velocity of 4.0 ± 0.1 mm/min (*n* = 7 slices from 4 rats). The maximal amplitude of negative LFP shift during AD was observed in the superficial layers (13.8 ± 0.8 mV at a depth of 400 μm from the cortical surface; *n* = 7; Figures 2C,D). In L4 and L5/L6 the amplitude of AD was 12.0 ± 0.6 and 10.7 ± 0.7 mV, respectively (*n* = 7). The OIS profile of AD was remarkably similar to that of the electrophysiological response including an initial onset in L4 and vertical delays in the supra- and infragranular layers, the speed of vertical propagation, and a maximal amplitude of light transmittance increase of 38.5 ± 10.3% (*n* = 7) attained in L2/3 and smaller change in deeper layers (Figures 2E,F).

### Patterns of AD initiation and propagation

**Figure 3:**
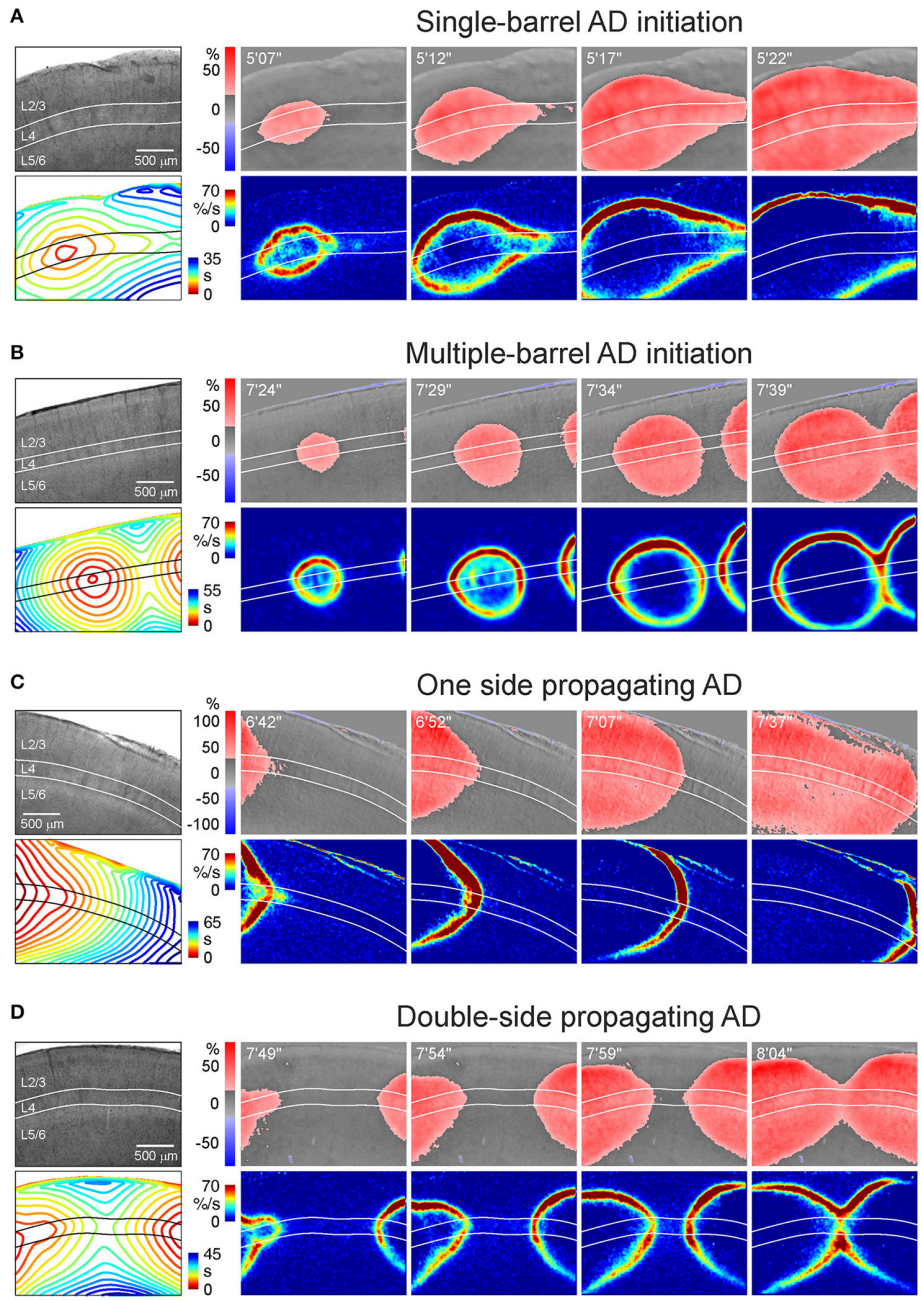
Various patterns of AD initiation and propagation in barrel cortex. **(A)** Microphotograph of a barrel cortex slice in DIC-IR (left) and OIS snapshots at different time points after OGD induction. Below, the corresponding AD fronts are presented as the OIS first derivative. Bottom left panel shows time color coded AD front contours plotted at 3 s intervals. Note that AD is initiated in one barrel and further propagates preferentially through L4. **(B)** Similar example of AD which is initiated in a barrel located in the middle of the imaging window and which collides with another AD wave arriving from another locus on the right of the imaging window. **(C)** Example of AD propagating preferentially through layer 4 from the left side of the imaging window. **(D)** Example of AD propagating from the left and right sides and colliding in the middle of the imaging window in L4.

OIS imaging revealed variability of the AD initiation and propagation patterns, which could be classified in four main groups (Figure 3 and Videos 1–4):

1. *Single-barrel AD initiation* (Figure 3A; Video 1). AD emerges in one barrel within the imaging window and spreads concentrically. The AD spread is often anisotropic with a preferential horizontal propagation along L4 forming a characteristic “bird head” OIS image. This pattern was observed in 11 of 30 slices.
2. *Multiple-barrel AD initiation* (Figure 3B; Video 2). AD emerges in two (or more) barrels within the imaging window. AD fronts move concentrically and collide first in L4 and then in the superficial and deep layers (*n* = 6 of 30 slices).
3. *One side propagating AD* (Figure 3C; Video 3). AD originates on one side of slice but outside of the imaging window and spreads through the slice with a preference to L4 (*n* = 8 of 30 slices).
4. *Double-side propagating AD* (Figure 3D; Video 4). AD emerges on two sides of slice outside of the imaging window. Two AD waves move toward each other with a preference to the L4 and collide similarly to the multi-barrel initiation pattern (*n* = 5 of 30 slices).

These results indicate that despite variety in the site of OGD-induced AD initiation in a slice, preference of AD to L4 is a hallmark of all initiation and propagation AD patterns. The rate of AD propagation along L4 was 1.7 ± 0.1 mm/min (*n* = 18 slices from 8 rats) which is consistent with the rate of AD and SD propagation in slices and in the intact brain *in vivo* (Nedergaard, 1996; Basarsky et al., 1998; Joshi and Andrew, 2001). In the cases of single-barrel AD initiation the rate of medial AD propagation along L4 was of 1.8 ± 0.1 mm/min that was not different from the rate of lateral AD propagation of 1.7 ± 0.1 mm/min (*n* = 11 slices from 4 rats, *p* = 0.76).

**Figure 4:**
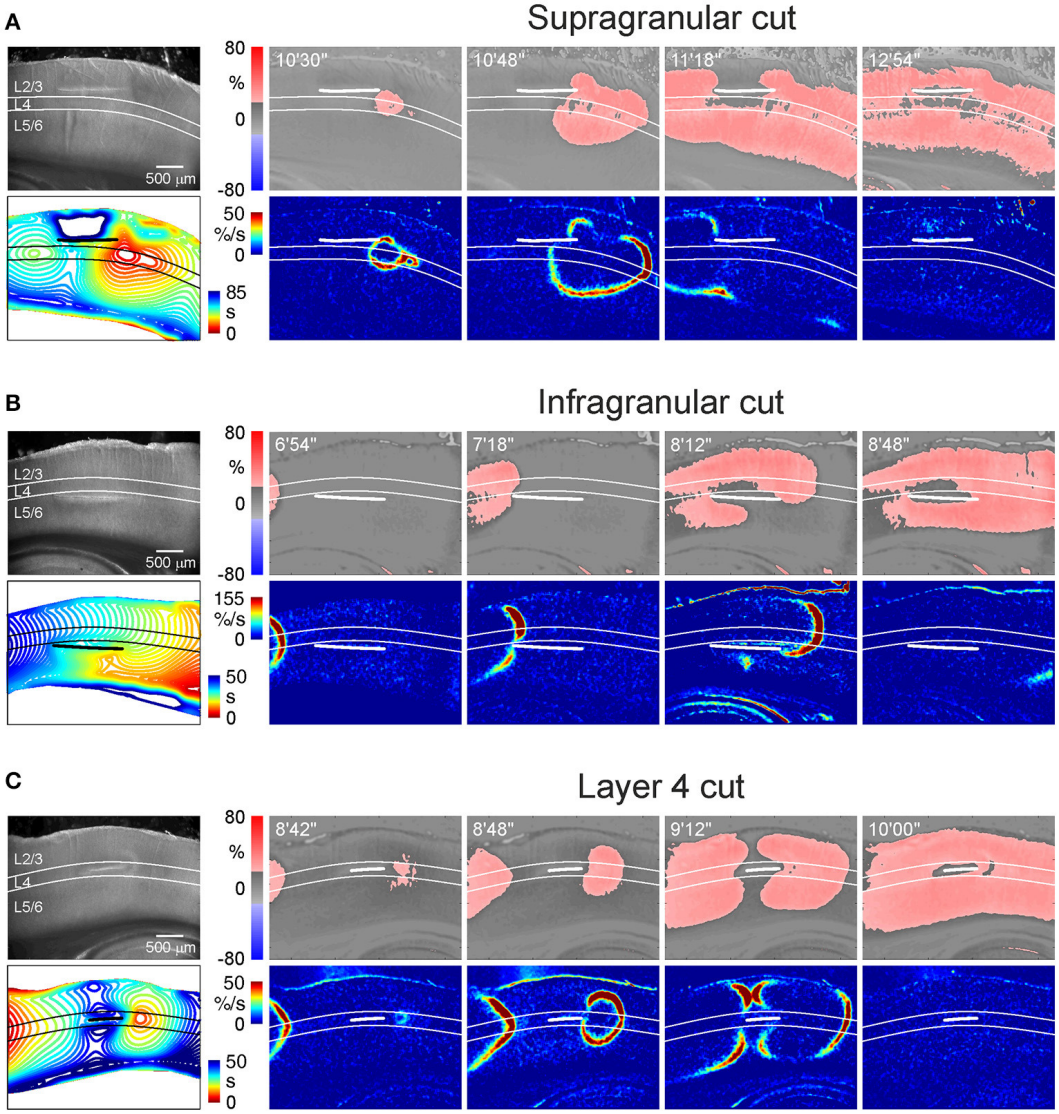
AD propagation after surgical cut above, below and through L4. **(A–C)** Example microphotographs of the barrel cortex slices in DIC-IR (left) with the cuts made above **(A)**, below **(B)**, and through **(C)** the layer 4 and OIS snapshots at different time points after OGD induction. Below, the corresponding AD fronts are presented as the OIS first derivative. Bottom left panel shows time color coded SD front contours plotted at 3 s intervals. Note that AD waves invade supragranular and infragranular layers in all cases but AD emerges earlier if connection with L4 is preserved. AD propagation to L2/3 above the cut on panel **(A)** was too slow and is truncated on the contour map. See Video 5 for the entire AD wave in this experiment.

We next addressed a question of whether AD in L4 is a necessary condition for emergence of AD in the supragranular and infragranular layers. In this aim, we explored OGD-induced AD after surgical cuts made above, below and through the L4 (Figure 4 and Videos 5–7). We found that AD efficiently invaded supragranular and infragranular layers even after disconnection from L4, with an AD front moving horizontally around the cuts thus indicating that both superficial and deep layers are capable of generating AD independently from L4. We further calculated the time difference between AD in the surface and deep layers at the areas vertically aligned to the middle of the cut. When the cut was made above L4, AD arrived to L2/3 88 ± 19 s later than to L5/6 (*n* = 4). When the cut was made below L4, AD in L2/3 emerged 49 ± 6 s earlier than in L5/6 (*n* = 5). Thus, AD was generated earlier in the layers maintaining connection with L4 than AD in the layers disconnected from L4. With the cut made through L4, the time delay between AD in L2/3 and L5/6 reduced to 12 ± 4 s (*n* = 4). Together, these results indicate that AD in L4 is not a necessary condition for AD in the surface and deep layers, where AD can propagate horizontally. However, vertical AD vector originating from L4 accelerates AD in the supragranular and infragranular layers maintaining their connection with L4.

### Elevated extracellular potassium concentration accelerates the AD onset

**Figure 5:**
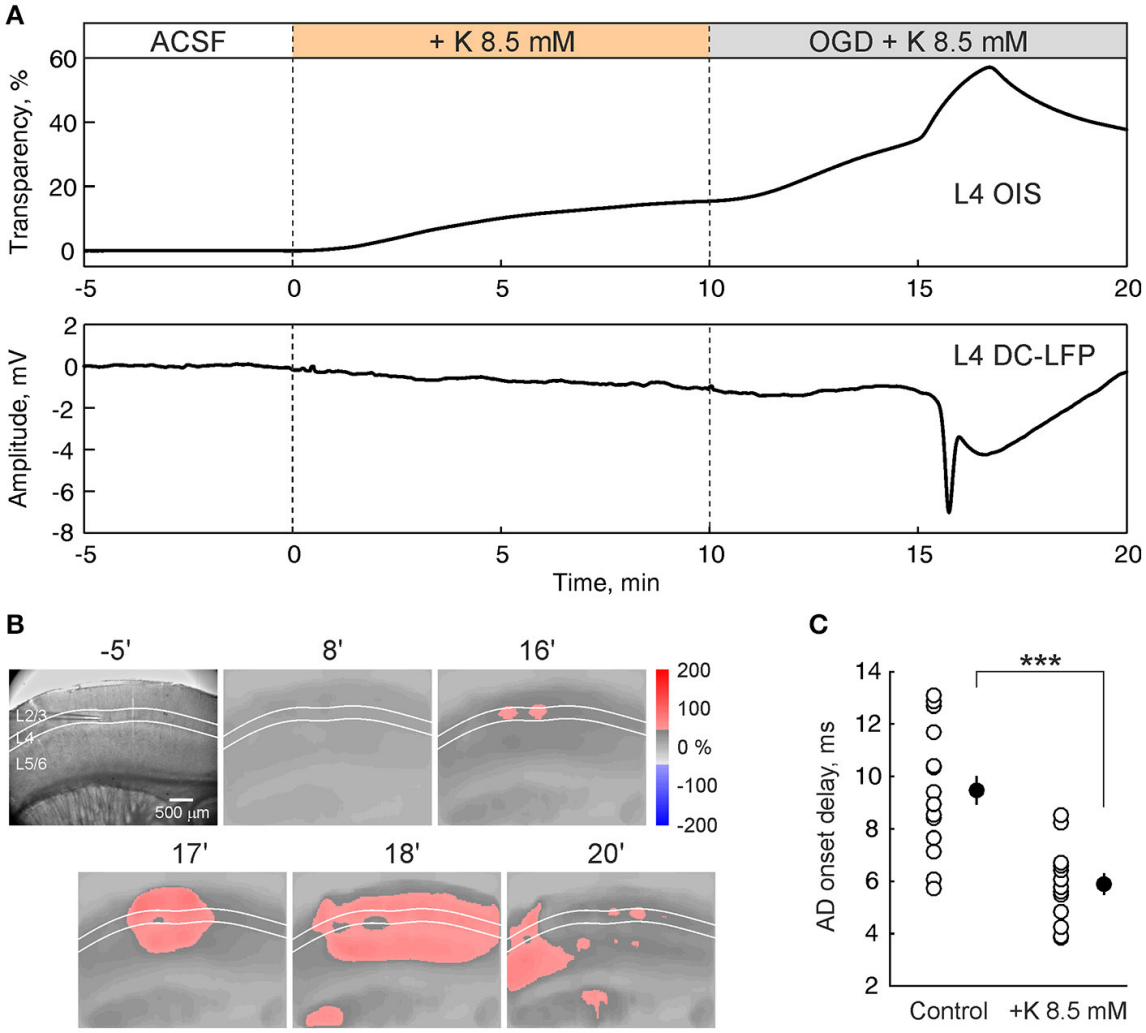
Elevation of extracellular potassium accelerates the onset of anoxic depolarization. **(A)** OIS from L4 (top trace) and nearby L4 DC-coupled LFP (bottom trace) during superfusion with ACSF with elevated (from 3.5 to 8.5 mM) potassium concentration and after OGD induction. **(B)** Corresponding microphotograph (left) and OIS snapshots at different time points after OGD induction. **(C)** Group data on AD onsets in control ACSF (*n* = 17) and in the ACSF with potassium concentration elevated to 8.5 mM (*n* = 13). Each white circle corresponds to one slice and black circles show the mean ± SE. Note that AD onset is accelerated almost two-fold after elevation of extracellular potassium concentration. ^***^p < 0.001.

Hyperactivity compromises the metabolic state of the tissue under OGD-conditions and accelerates the AD onset in hippocampus (Dzhala et al., 2000). With the aim of exploring the effect of increased activity on the OGD-induced AD in the barrel cortex, we elevated extracellular potassium concentration in ACSF from 3.5 to 8.5 mM. The high-potassium solution itself induced a slight increase in light transmittance and a negative shift in the LFP baseline in L4 (Figure 5A). Further superfusion with high-potassium/OGD solution evoked AD with a delay of 5.9 ± 0.4 min (*n* = 13 slices from 5 rats), that was almost two-fold quicker (*p* < 0.001) than AD evoked by OGD in normal potassium conditions (9.5 ± 0.5 min; *n* = 17 slices from 8 rats) (Figure 5C). In the high-potassium/OGD solution the negative LFP shift of 8.5 ± 0.6 mV (*n* = 13) in L4 was similar to those in normal potassium conditions (*p* > 0.05) while the increase in light transparency of 19.8 ± 1.0% dI/I (*n* = 13) was less than in normal conditions (*p* < 0.05), due to the progressive increase of dI/I before AD which occurs in elevated potassium conditions (Figure 5A). OIS imaging revealed that preference of AD initiation and propagation in L4 was maintained under conditions of elevated potassium (Figure 5B).

### Highest metabolic activity in L4

**Figure 6:**
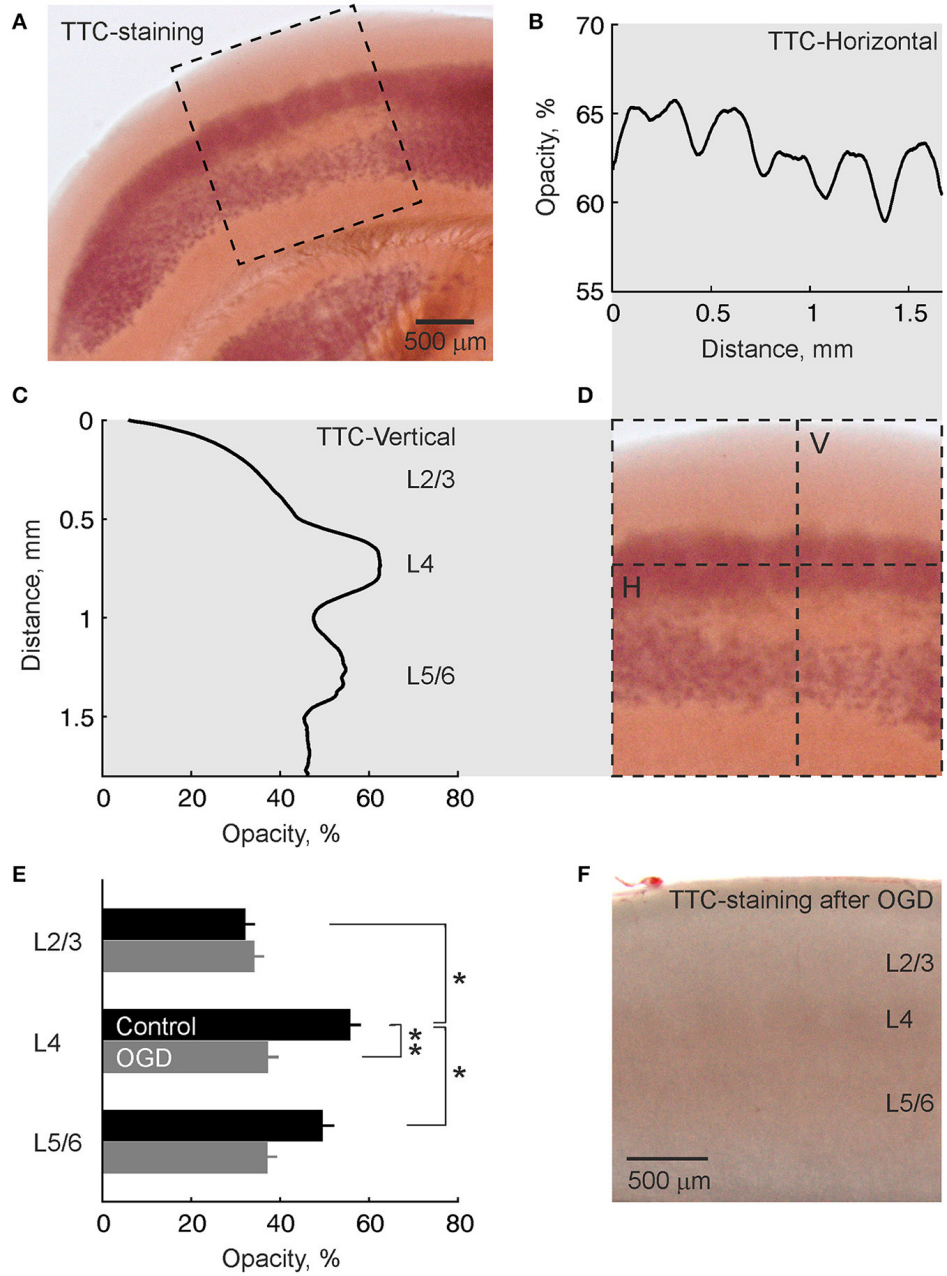
TTC-staining of a live slice of the barrel cortex shows highest metabolic activity in L4. **(A)** Microphotograph of a slice of barrel cortex stained with TTC. **(B–D)** Intensity of TTC-staining within a region outlined by dashed box on panel **(A)** in **(B)** horizontal projection along the L4 (indicated by H-line on panel **D**) and **(C)** vertical projection across cortical layers of a barrel column (indicated by V-line on panel **D**). Note the horizontal barrel staining pattern and maximal staining of L4 in vertical projection. **(E)** Group data on the intensity of TTC-staining of different cortical layers in control slices (*n* = 7) and slices after OGD exposure (*n* = 9). ^*^p < 0.05; ^**^p < 0.01. **(F)** Microphotograph of a TTC-stained slice of barrel cortex after OGD exposure. Note weak non-specific staining compared with the live slice.

Preferential initiation and propagation of AD in the barrels may involve the higher metabolic demand of barrels and therefore their higher vulnerability to metabolic deprivation. We explored this hypothesis using 2,3,5-triphenyltetrazolium chloride (TTC) staining of live slices of the barrel cortex. As shown on Figures 6A,D, TTC most intensively stained L4. Quantification of TTC-staining along the horizontal projection in L4 revealed peaks in TTC-staining corresponding to neighboring barrels (Figure 6B). In the vertical projection across cortical depth, TTC staining peaked at the L4 depth (Figure 6C). A second, less intense peak was found ~0.5 mm deeper at the L5B/L6A border (Figure 6C). Cross-layer comparisons revealed significantly higher TTC-staining of L4 compared to L2/3 (*p* < 0.05) and L5/6 (*p* < 0.05) (*n* = 7 slices from 4 rats; Figure 6E). Slices that had been exposed to OGD for 30 min and reperfused with normal ACSF for 2 h displayed only weak non-specific staining (Figure 6F). TTC-staining of L4 in OGD-exposed slices revealed no difference with L2/3 (*p* > 0.05) and L5/6 (*p* > 0.05) staining and was significantly lower compared to control slices (*p* < 0.01; *n* = 9 slices from 4 rats) (Figure 6E).

## Discussion

The principal conclusion emerging from the present study is that different layers of the barrel cortex differ in their propensity to AD and that L4 is the most prone to AD. This conclusion is supported by electrophysiological recordings and OIS imaging indicating that OGD-induced AD is preferentially initiated in, and preferentially spreads through L4. We also found that enhanced L4 susceptibility to OGD correlates with the highest metabolic activity, in L4, revealed with TTC staining of live slices.

Anisotropy is a characteristic feature of the heterogeneous incidence and horizontal spread of cortical SD *in vivo* (Kaufmann et al., 2017). Previous studies in non-identified neocortical areas revealed anisotropy of AD and SD across cortical layers with a “tropism” to the superficial layers 2/3 (Basarsky et al., 1998; Joshi and Andrew, 2001; Kaufmann et al., 2017). However, our findings indicate that in the barrel cortex, which contains large barrels and the thickest L4 of all the cortical regions, AD is initiated and preferentially propagates via L4. Onset and preferential propagation of AD in L4 was evidenced by the earliest onset of the negative LFP DC shift and the earliest increase in optical transparency in barrels during the OGD-induced AD. Multisite LFP recordings and simultaneous OIS imaging revealed vertical spread of AD from the L4 to the superficial and deep layers within a column. OIS recordings also enabled us to assess two-dimensional spatial-temporal AD dynamics in the barrel cortex slices revealing a variety of AD initiation and propagation patterns, yet with a common delimiter of the highest proneness of L4 to AD. While AD primarily originated in L4 in the barrel cortex, L4 appeared to be not necessary for the emergence of AD in the supragranular and infragranular layers, however. Indeed, our experiments with surgical cuts above, below and through the L4 revealed that AD may propagate through these layers horizontally around the cuts, although at lower speed. This indicates that supra- and infragranular layers are capable of generating AD independently from the L4. Yet, early ignition of AD in L4 is important for driving AD in the supra- and infragranular layers in the intact slice.

Various factors have been suggested to explain anisotropy of SD and AD (Herreras and Somjen, 1993; Somjen, 2001; Canals et al., 2005; Kaufmann et al., 2017). High neuronal density in L2/3 has been hypothesized to promote a mutual promotion of depolarization and potassium release and accumulation, making these layers more prone to AD (Joshi and Andrew, 2001). In the barrel cortex, the highest neuronal density is observed in L4, where it attains 124 thousand neurons/mm^3^ compared to 102 and 86 thousand neurons/mm^3^ in L3 and L2, respectively (Meyer et al., 2010). Thus, our findings of the preferential initiation and spread of AD in L4 of the barrel cortex are consistent with the “neuronal density” hypothesis. Interestingly, SD and AD preferentially arise in and propagate through the whisker barrel region of parietal sensory cortex *in vivo* (Bogdanov et al., 2016; Kaufmann et al., 2017). Because the barrel cortex contains large barrels and the thickest L4 of all the somatosensory cortical regions, the elevated proneness of L4 to AD as revealed in the present study may also explain high proneness of the barrel cortex to AD.

Our observations of heterogeneous TTC-staining in different cortical layers with the maximum in L4 suggest that elevated metabolic demand could also be a factor contributing to the particular susceptibility of this layer to AD. TTC staining intensity is determined by the metabolic activity of mitochondrial dehydrogenases, which enzymatically convert colorless TTC to red formazan (Goldlust et al., 1996). The elevated L4 metabolic activity revealed with TTC-staining is consistent with the highest density of a mitochondrial enzyme cytochrome oxydase and elevated number of mitochondria in L4 of the barrel cortex where they reside mainly in dendrites and axonal terminals (Wong-Riley and Welt, 1980). Considerable evidence indicates that elevation of metabolic debt strongly aggravates ischemic insults. Indeed, various factors increasing the metabolism such as an increase in neuronal activity caused by adenosine A1 receptor antagonists, blockers of GABA(A) receptors and potassium channels (Dzhala et al., 2000), elevation of extracellular potassium as in the present study or elevated temperature (Joshi and Andrew, 2001) strongly accelerate the AD onset. Thus, due to elevated metabolic activity, L4 neurons are most likely to quickly lose ATP, depolarize and ignite AD in metabolically-compromised conditions. The question then arises: why the metabolic activity is highest in L4 barrels? Although the underlying mechanisms are unknown, it could be suggested that it involves a particular cytoarchitectonic and synaptic barrel organization. Indeed, densely packed excitatory and inhibitory neurons form a highly interconnected network in L4 barrels (Feldmeyer et al., 1999; Lefort et al., 2009; Valiullina et al., 2016) that may impose a higher metabolic charge to equilibrate the ionic disturbances caused by the activity in this layer.

Thus, in the present study we have shown that different cortical layers differ in their sensitivity to metabolic insult with L4 being the most prone to AD initiation and preferential propagation. Elevated ongoing metabolic demand of L4 could be a factor contributing to this enhanced sensitivity of L4 to OGD. Our findings also support rationale of the strategies aimed to reduce the metabolic demand as an approach to alleviate ischemic brain damage.

## Author contributions

RK conceived the project. EJ and MM performed the experiments. AN, MM, EJ, AG, and MS analyzed the data. RK wrote the paper.

### Conflict of interest statement

The authors declare that the research was conducted in the absence of any commercial or financial relationships that could be construed as a potential conflict of interest.

## Supplementary material

Video 1 Single-barrel AD initiation pattern (related to Figure 3A). Left, OIS and right, AD front. 5× playback speed.

Video 2 Multiple-barrel AD initiation (related to Figure 3B). Left, OIS and right, AD front. 5× playback speed.

Video 3 One side propagating AD (related to Figure 3C). Left, OIS and right, AD front. 5× playback speed.

Video 4 Double-side propagating AD (related to Figure 3D). Left, OIS and right, AD front. 5× playback speed.

Video 5 AD propagation in a slice with a cut above L4 (related to Figure 4A). Left, OIS and right, AD front. 5× playback speed.

Video 6 AD propagation in a slice with a cut below L4 (related to Figure 4B). Left, OIS and right, AD front. 5× playback speed.

Video 7 AD propagation in a slice with a cut through L4 (related to Figure 4C). Left, OIS and right, AD front. 5× playback speed.
